# Update on the integrated histopathological and genetic classification of medulloblastoma – a practical diagnostic guideline 

**DOI:** 10.5414/NP300999

**Published:** 2016-10-26

**Authors:** Torsten Pietsch, Christine Haberler

**Affiliations:** 1 DGNN Brain Tumor Reference Center, Institute of Neuropathology, University of Bonn Medical Center, Bonn, Germany,; 2 Institute of Neurology, and; 3 Comprehensive Cancer Center, Medical University of Vienna, Vienna, Austria

**Keywords:** WHO classification, medulloblastoma, immunohistochemistry, WNT, SHH, genetics

## Abstract

The revised WHO classification of tumors of the CNS 2016 has introduced the concept of the integrated diagnosis. The definition of medulloblastoma entities now requires a combination of the traditional histological information with additional molecular/genetic features. For definition of the histopathological component of the medulloblastoma diagnosis, the tumors should be assigned to one of the four entities classic, desmoplastic/nodular (DNMB), extensive nodular (MBEN), or large cell/anaplastic (LC/A) medulloblastoma. The genetically defined component comprises the four entities WNT-activated, SHH-activated and *TP53 *wildtype, SHH-activated and *TP53 *mutant, or non-WNT/non-SHH medulloblastoma. Robust and validated methods are available to allow a precise diagnosis of these medulloblastoma entities according to the updated WHO classification, and for differential diagnostic purposes. A combination of immunohistochemical markers including β-catenin, Yap1, p75-NGFR, Otx2, and p53, in combination with targeted sequencing and copy number assessment such as FISH analysis for *MYC* genes allows a precise assignment of patients for risk-adapted stratification. It also allows comparison to results of study cohorts in the past and provides a robust basis for further treatment refinement.

## Introduction 

Since the last version of the WHO classification of tumors of the CNS was published in 2007, knowledge on genetic alterations and biological features of medulloblastoma has rapidly increased by (epi)genome- and transcriptome-wide studies. In particular, RNA expression studies and DNA methylation profiling have led to the identification of “biological” variants of medulloblastoma defined by predominant signalling pathways and DNA methylation patterns associated to their cellular origin [24]. These molecular subgroups overlap with histological features in some cases but are discordant in others. While the WHO classification 2007 relied on histological features only, the challenge of its update in 2016 was to integrate meaningful genetic/biological information to enable a more precise classification of medulloblastoma without disrupting the continuity of the classification system. Continuity is especially needed for (1) longitudinal comparisons of outcome data of clinical studies, (2) the comparison of associated research data, and (3) a reliable basis for epidemiological information on the incidence of the disease entities. 

This challenge was elegantly resolved by the introduction of the integrated diagnosis concept in the WHO classification 2016 [17]. Most CNS tumor entities are still classified on only histopathological features, but in medulloblastoma, the definition of disease entities requires an integration of additional molecular information. This approach improves the definition of the medulloblastoma entities, reduces the interobserver variability in diagnostics, and allows a better selection of patients for treatment stratification as well as improved prediction of treatment response and prognosis. By this approach, the traditional histological diagnosis (e.g., “classic medulloblastoma”) and the histological grade of malignancy (“WHO grade IV”) are combined with defined molecular genetic/biological features (e.g., “WNT activation”). In daily diagnostic practice, the histological part of the diagnosis can be made in the same short time frame as before, the molecular diagnostic part requires additional tests which might need more time for analysis, but finally, an integrated diagnosis e.g., “classic medulloblastoma, WNT activated, WHO grade IV” can be signed out, now precisely describing a well-defined disease entity. 

Some limitations for the integration of molecular characteristics come from a technical aspect. The methods employed for proper classification of medulloblastomas have to be (1) available or accessible in most neuropathological units worldwide and (2) certifiable as diagnostic test systems. In the WHO classification 2016 no recommendation for the use of specific test systems or methods is given, but care was taken that the implementation of molecular markers for classification is possible in daily diagnostic practise in most laboratories. 

## WHO classification of medulloblastomas – histologically and genetically defined 

Although the five histological entities of medulloblastomas according to the 2007 classification could be validated as prognostic and useful in the stratification of patients for risk-adapted treatment in clinical studies, in particular in young children, the histological subtyping had several limitations. For example, interobserver variability in the assessment of certain histological features caused problems, e.g., the differentiation of “true” desmoplastic medulloblastoma variants vs. cases with desmoplastic (reactive) changes due to superficial growth, or the differentiation between large cell and anaplastic medulloblastoma variants in cases showing both cytological components. The histological subtype was often found related to certain genetic/biological features of the tumors but considered to be not as distinctive as a definition by mRNA expression or DNA methylation signatures. To improve the precision of medulloblastoma diagnostics, the histological typing is now combined with genetic information to allow for an informative diagnosis. In the histological part, only one adaptation was made in the WHO classification 2016: the large cell and anaplastic medulloblastomas are now jointly diagnosed as large cell/anaplastic (LCA) medulloblastomas because it was felt difficult to differentiate these rare entities that often show a mixed cellular composition. This term had also been used in the 2000 WHO classification before. The definitions of the *histologically defined* medulloblastoma variants did otherwise not change significantly compared to the WHO classification 2007 (Table 1). 

Regarding the *genetically defined* component of the diagnosis, four main entities were newly defined (Table 1) [4]. 

One entity is “medulloblastoma, WNT-activated”. These tumors cannot be identified on hematoxylin-eosin (H & E)-stained sections alone; most of them have classic morphology but immunohistochemically show nuclear accumulation of β-catenin protein as surrogate biomarker for WNT activation caused by *CTNNB1* activating mutations or – rarely – mutations in *APC* or other genes encoding components of the WNT signaling pathway [6, 11, 12]. The precise identification of these tumors is important because of their excellent prognosis in the pediatric age (< 16 years) and inclusion of patients into ongoing therapeutic trials aiming to prove that reduction of treatment intensity is possible in these patients (e.g., the European SIOP PNET5 medulloblastoma trial). In the setting of such clinical trials, it is widely recommended to use two independent methods for reliable identification of these patients such as immunohistochemistry for β-catenin and sequencing of *CTNNB1* exon 3 or alternative methods (Nanostring RNA profiling, methylation classifiers) [7]. 

Tumors showing mRNA expression and DNA methylation profiles suggesting activation of sonic hedgehog (SHH) signaling are considered to represent two very different disease entities, depending on the *TP53 *genetic status [13, 26]. Therefore, two genetically defined entities are “medulloblastoma, SHH-activated and *TP53*-mutant” and “medulloblastoma, SHH-activated and *TP53*-wildtype”. The latter occur mostly in adolescents/adults and young children who have a good prognosis if adequately treated. In contrast, *TP53*-mutant SHH medulloblastomas occur in older children and have a dismal prognosis [26]. SHH activation is caused by mutations in *PTCH1*, *SUFU*, *SMO,* or other components of the SHH signaling pathway [20]. Fortunately, SHH activation can be reliably assessed by different methods including a panel of antibodies against SHH target proteins (see below) [5, 14]. On the other hand, if a SHH-activated tumor is identified, the *TP53* genetic status has to be determined for a precise classification. Proper identification of SHH activation is also important because a significant fraction of young children with SHH-activated medulloblastomas have underlying germ-line mutations of *PTCH1* or *SUFU* (Gorlin syndrome) [2]. These patients and their families should be offered genetic counselling. The same is true for patients suffering from *TP53*-mutant SHH medulloblastomas indicating possible *TP53* germline mutations (Li-Fraumeni syndrome) or other germline defects. 

The fourth genetically defined entity represents the majority of medulloblastomas lacking either WNT or SHH pathway activation (non-WNT/non-SHH medulloblastomas). These tumors seem to lack recurrent mutations but show frequent chromosomal copy number alterations such as isochromosome 17q. They can be further subdivided with DNA methylation profiling or mRNA expression studies in “group 3” and “group 4” medulloblastomas. *MYC* amplification is frequently found in young children with group 3 tumors, and metastatic disease at diagnosis and is associated with a very dismal outcome. Group 3 and 4 variants have so far only been considered as provisional subentities because it is not absolutely clear if they represent distinct diseases or variants of a single entity. 

In adult patients, only three biological medulloblastoma subgroups have been identified: WNT, SHH, and group 4 [22]. In contrast to children, WNT tumors could not be associated with improved survival and patients with group 4 tumors have been reported to have a dismal outcome as compared to patients with SHH and WNT tumors [25]. 

All combinations between histological and genetic parts of the medulloblastoma classification scheme are theoretically possible, but there are frequent associations. For example, most WNT-activated medulloblastomas are of classic histology (as are most non-WNT/non-SHH medulloblastomas), most SHH-activated cases with *TP53*-mutation show an anaplastic phenotype, and almost all desmoplastic/nodular medulloblastomas and those with extensive nodularity are SHH-activated [20]. The term “medulloblastoma, not otherwise specified (NOS)” should be restricted to cases with insufficient material for further analysis or inconclusive results of molecular testing. In summary, the concept of an integrated diagnosis is used for WHO classification of medulloblastomas and allows a precise assignment of patients for risk-adapted stratification. It also allows comparison to results of study cohorts in the past and provides a robust basis for further treatment refinement. 

## Histopathological assessment in neuropathological practice 

**Figure 1.**
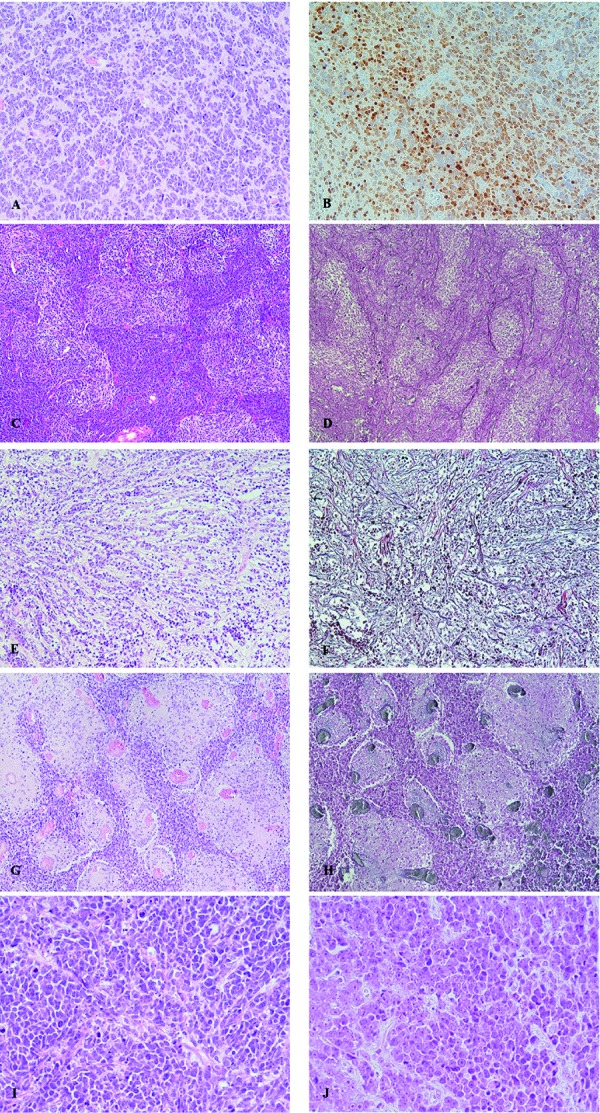
Histologically defined entities of medulloblastoma. A: Classic medulloblastoma with (B) strong NeuN expression in preexisting granule cells and weaker expression in tumor cells (NeuN); C: desmoplastic/nodular medulloblastoma with (D) reticulin fibers in internodular areas (reticulin stain); E: classic medulloblastoma without pale nodular areas but with (F) desmoplastic reaction due to leptomeningeal invasion (reticulin stain); G: classic medulloblastoma with pale nodules but (H) without desmoplasia (reticulin stain); I, J: large/cell anaplastic medulloblastoma with (I) severely anaplastic nuclei with nuclear moulding/wrapping and frequent mitotic and apoptotic figures; J: large round cells with prominent nucleoli.

For proper definition of the histopathological component of the medulloblastoma diagnosis, the tumors should be assigned to one of the four entities classic, desmoplastic/nodular (DNMB), extensive nodular (MBEN) or large cell/anaplastic (LC/A) medulloblastoma (Figure 1). One important issue is the differentiation of “true” desmoplastic/nodular medulloblastoma variants (Figure 1C, D) vs. cases with desmoplastic (reactive) changes due to superficial growth (Figure 1E, F), and vs. cases showing nodular appearance but no reticulin fibers (Figure 1G, H) [18]. For correct identification of desmoplastic/nodular medulloblastoma both H & E staining and silver impregnation (reticulin staining) is necessary. Reticulin fibers must be present ensheating nodular areas lacking such argyrophilic fibers. A reactive desmoplastic reaction, which may occur in all types of medulloblastomas does not result in a regular nodular pattern as seen in DNMB. On the other hand, a tumor qualifies for the diagnosis of DNMB if the characteristic pattern is present in restricted areas only, even if other parts lack a desmoplastic/nodular histology. Nodular medulloblastoma entities do usually not contain neuroblastic rosettes. MBEN is regarded as being closely related to DNMB but has to contain larger islands with differentiated neurocytic cells. These should be predominant and can be illustrated by strong expression of NeuN antigen. MBEN as well as DNMB must show a SHH activation according to the revised WHO classification 2016 [20]; therefore, immunohistochemistry for SHH target proteins can be used for the differential diagnosis of nodular entities vs. classic medulloblastoma (see below). For the diagnosis of large cell/anaplastic medulloblastoma, a severely anaplastic (Figure 1I) or a large cell component (Figure 1H) or a mixture of both should be predominant (more that 50% of the tumor area). Cytological anaplasia is reflected by increased nuclear variability, frequent mitoses/apoptoses and nuclear wrapping, while the large cell cytology is characterized by cells with round larger nuclei with singular prominent nucleoli. The latter cells frequently show a dot-like immunoreactivity with antibodies against synaptophysin [21]. 

## Practical approach to the assessment of the genetic component of medulloblastoma 

For proper identification of the four genetic entities according to the WHO classification, a combination of immunohistochemistry and genetic assays are very helpful. 

**Figure 2.**
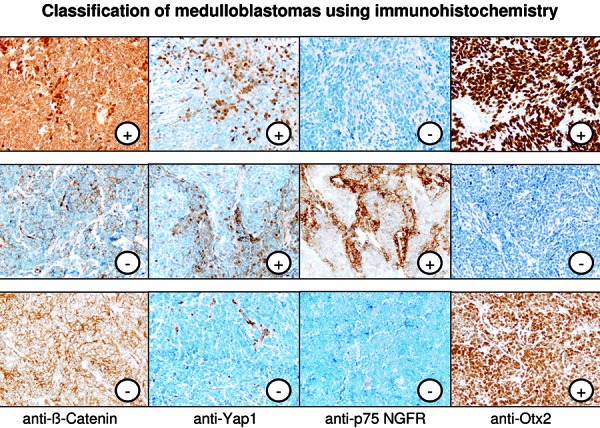
Characteristic immunophenotype of WNT-activated (upper panel), SHH-activated (middle panel) and non-WNT/non-SHH medulloblastomas (lower panel).

The WNT-activated medulloblastomas (~ 10% of all medulloblastoma), the two medulloblastoma entities with SHH activation (with or without *TP53 *alteration, together ~ 30% of all medulloblastomas), and the non-WNT/non-SHH medulloblastomas can be securely differentiated by a set of immunohistochemical markers (Table 2) (Figure 2), namely β-Catenin, Yap1, p75NGFR (or Gab1), and Otx2. WNT medulloblastomas show nuclear accumulation of β-catenin protein in addition to Yap1 immunoreactivity in tumor nuclei and express Otx2 [4, 14]. SHH-activated medulloblastomas express specific target proteins such as p75NGFR and Gab1, share expression of nuclear Yap1 with the WNT medulloblastoma, but lack Otx2 expression. Non-WNT/non-SHH medulloblastomas finally express Otx2 but lack the other markers such as Yap1 (which is only expressed in endothelial cells in this tumor type serving as internal control (Figure 2) and nuclear β-catenin. Recently produced batches of the Gab1 antibody do not seem to deliver reliable staining results, and we therefore recommend the use of Otx2 and p75NGFR instead. 

Immunohistochemistry for p53 should be performed at least in all SHH-activated tumors, because p53 accumulation strongly indicates the SHH-activated *TP53-*altered medulloblastoma entity [26]. Most of these tumors show cytological anaplasia, at least focally. In SHH-activated tumors, *TP53 *should be sequenced with validated methods in certified laboratories to clearly differentiate between the SHH-activated medulloblastoma entities with and without *TP53 *alteration because of important consequences for treatment decisions and possible germ line alterations. It is a matter of debate if the sequencing of SHH-activated medulloblastomas can be restricted to tumors which show p53 accumulation (> 5% of nuclei), and/or signs of cytological anaplasia or if all SHH-activated medulloblastoma should be sequenced. Tabori et al. [23] found a 100% sensitivity of p53 accumulation to predict *TP53 *mutations in medulloblastomas. The age distribution of SHH-activated, *TP53*-altered cases shows a peak in the school-age so that SHH-MB tumors in these children should be carefully analyzed [13]. 

Most of the non-WNT/non-SHH medulloblastomas can be further subdivided in the provisional “group 3” and “group 4” variants by expression or methylation profiling. Although it has been suggested in the literature that group 3 and 4 tumors can be distinguished by immunohistochemical stainings [19], this could not be confirmed [1, 10], and to date no robust simple technologies (e.g., immunohistochemical methods) are available for their precise distinction. Even with array-based analytical methods, there is a “grey zone” between groups 3 and 4, with tumors switching groups if different algorithms for data analysis are employed. In pediatric patients, “group 3” non-WNT/non-SHH medulloblastomas contain standard-risk medulloblastomas (not behaving differently from “group 4” patients) as well as *MYC*-amplified tumors showing mostly a very poor prognosis. *MYC* amplification is considered an important prognostic biomarker within the non-WNT/non-SHH medulloblastomas [21] but not as a diagnostic marker defining an own entity. 

In Europe, multinational multicenter trials for the treatment of children with medulloblastoma, such as the standard risk medulloblastoma SIOP-PNET5 medulloblastoma trial, have set a framework for the establishment of national neuropathological reference centers for central review and classification of medulloblastomas in the last years. In these centers, validated methods have been established and harmonized. However, all immunohistochemical tests as well as genetic analyses should be validated in each diagnostic neuropathological unit to allow secure assignment of medulloblastoma patients according to the revised WHO classification 2016. 

## MYC and MYCN FISH analyses 

**Figure 3.**
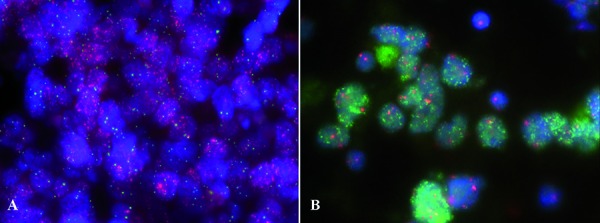
FISH showing representative tumors with (A) *MYC* amplification (*MYC* = red, CEP8 = green) and (B) *NMYC* amplification (*MYCN* = green, CEP2 = red).

*MYC* and *MYCN* amplification (Figure 3) have been described as important negative prognostic factors in pediatric medulloblastoma [15], and the negative impact of *MYC* amplification could be confirmed in adults [9]. Several methods can be used to detect gene amplifications including interphase FISH, CGH/SNP/molecular inversion profiling (MIP)/methylation arrays. iFISH analysis is considered the “gold-standard” for analysis of the *MYC* and *MYCN* copy number status in medulloblastomas in neuropathological practice. It allows a copy number estimation for specific genes on a single-cell level, detection of intratumoral heterogeneity, and requires only few tissue amounts of either FFPE tissue. As failure rates of iFISH on FFPE tissue in up to 15% of the cases are common, touch preparations of unfixed or frozen tumor tissue are recommended. In the majority of amplified cases, the vast majority of tumor cells harbor the amplification. Yet, a small number of tumors may also show only focal/patchy amplification. In the SIOP PNET5 study the cut-off for amplification is ≥ 5%, (in 200 non-overlapping nuclei) yet, it needs to be clarified whether patients with focal/patchy amplification have the same poor prognosis as patients with widespread amplification. 

## Differential diagnostic considerations in neuropathological practice 

In general, medulloblastomas of the different entities express neural markers such as Map2 and CD56, and also frequently the synaptic protein synaptophysin. Other undifferentiated tumors of the cerebellum should be excluded. In young children, AT/RT, ETMR, and CPC should be considered in particular. Immunohistochemistry with antibodies against Ini1/Smarcb1, Lin28, EMA, and cytokeratin is very helpful in this regards since medulloblastomas lack loss of Ini1 and do not show expression of the other antigens. Anaplastic ependymomas may be detected by staining with antibodies against EMA and GFAP. Undifferentiated gliomas can be identified by staining with antibodies against GFAP and Olig2. Diffuse midline gliomas frequently harbor a H3K27 mutation and can be detected with a specific antibody. Sarcomas are identified by silver impregnation methods (reticulin staining). 

In adults, metastatic disease of melanomas or small cell/neuroendocrine carcinomas should be considered and immunohistochemistry for cytokeratins and melanotic antigens (HMB-45/MelanA) may be appropriate. 

In summary, widely accessible and robust methods are available to allow a precise diagnosis of medulloblastoma entities according to the revised WHO classification of brain tumors 2016, and for differential diagnostic purposes. Novel technologies including genome-wide methylation arrays or sequencing techniques are now used in many neuropathological units for research projects. However, these methods have to be thoroughly validated and qualified for certification before they might be used as tools for clinical diagnostics in the future. 

## Conflict of interest 

The authors declare no conflict of interests. 

**Table 1.** Medulloblastoma is classified by an integrative diagnosis including a histologically as well as genetically defined compound.

*Medulloblastoma, histologically defined*
Medulloblastoma, classic
Medulloblastoma, desmoplastic/nodular
Medulloblastoma with extensive nodularity
Medulloblastoma, large cell/anaplastic
*Medulloblastoma, genetically defined*
Medulloblastoma, WNT-activated
Medulloblastoma, SHH-activated, TP53 mutated
Medulloblastoma, SHH-activated, TP53 wild-type
Medulloblastoma, non-WNT/non-SHH
Medulloblastoma, group 3 *
Medulloblastoma, group 4 *
Medulloblastoma, NOS**

*Provisional entity; **NOS (not otherwise specified) should only be used when no appropriate material is available for classification.

**Table 2.** Antibodies and FISH probes recommended for the analysis of medulloblastomas.

Antigen	Antibody/Clone	Supplier	Medulloblastoma subtype(s)	Reference
β-catenin	Mouse MAb/C14	Cell Marque, Rocklin, CA, USA	WNT (nuclear accumulation)	Ellison et al. 2011 [5]
P75-NGFR	Mouse MAb/NGFR5	Thermo, Runcorn, UK	SHH	Küchler et al. 2011 [14]
Gab1	Rabbit polyclonal Cat # 06-79	Merck-Millipore, Darmstadt, Germany	SHH	Ellison et al. 2011 [5]
Yap1	Rabbit Mab/D8H1X	Cell Signaling, Danvers, MA, USA	WNT and SHH	Ellison et al. 2011 [5]
Otx2	Mouse MAb/1H12C4B5	Thermo Fisher, Rockford, IL, USA	WNT and Non-WNTnon-SHH	De Haas et al. 2006 [2]
NeuN	Mouse MAb/A60	Merck-Millipore, Darmstadt, Germany	MBEN-SHH	Giangaspero et al. 2016 [7]
P53	Mouse MAb/DO-7	Dako, Hamburg, Germany	SHH-T53 altered WNT with TP53 alteration	Tabori et al. 2010 [23] Zhukova et al. 2013 [26]
Gene	FISH Probes	Supplier	MB subtype(s)	
MYC	Vysis LSI MYC/CEP8	Abbott, Wiesbaden, Germany	Non-WNT/non-SHH	
	MYC (8q24)/SE 8	Kreatech/Leica, Wetzlar, Germany		
MYCN	Vysis LSI N-MYC (2p24)/CEP 2	Abbott	SHH-p53 altered Non-WNT/non-SHH	
	MYCN (2p24)/AFF3 (2q11)	Kreatech		

## References

[1] Bien-Willner GA López-Terrada D Bhattacharjee MB Patel KU Stankiewicz P Lupski JR Pfeifer JD Perry A Early recurrence in standard-risk medulloblastoma patients with the common idic(17)(p11.2) rearrangement. Neuro-oncol. 2012; 14: 831–840. 22573308 10.1093/neuonc/nos086 PMC3379796

[2] de Haas T Oussoren E Grajkowska W Perek-Polnik M Popovic M Zadravec-Zaletel L Perera M Corte G Wirths O van Sluis P Pietsch T Troost D Baas F Versteeg R Kool M OTX1 and OTX2 expression correlates with the clinicopathologic classification of medulloblastomas. J Neuropathol Exp Neurol. 2006; 65: 176–186. 16462208 10.1097/01.jnen.0000199576.70923.8a

[3] Eberhart CG Cavenee WK Pietsch T Naevoid basal cell carcinoma syndrome. In: Louis DN, Ohgaki H, Wiestler OD, Cavenee WK (eds.) World Health Organization classification of tumors of the central nervous system. Revised 4th edition. IARC. 2016. p. 319-321.

[4] Ellison DW Giangaspero F Eberhart CG Haapasalo H Pietsch T Wiestler OD Pfister S Medulloblastomas, genetically defined. In: Louis DN, Ohgaki H, Wiestler OD, Cavenee WK (eds.) World Health Organization classification of tumours of the central nervous system. Revised 4th edition. IARC. 188-193. 2016.

[5] Ellison DW Dalton J Kocak M Nicholson SL Fraga C Neale G Kenney AM Brat DJ Perry A Yong WH Taylor RE Bailey S Clifford SC Gilbertson RJ Medulloblastoma: clinicopathological correlates of SHH, WNT, and non-SHH/WNT molecular subgroups. Acta Neuropathol. 2011; 121: 381–396. 21267586 10.1007/s00401-011-0800-8 PMC3519926

[6] Ellison DW Onilude OE Lindsey JC Lusher ME Weston CL Taylor RE Pearson AD Clifford SC β-Catenin status predicts a favorable outcome in childhood medulloblastoma: the United Kingdom Children’s Cancer Study Group Brain Tumour Committee. J Clin Oncol. 2005; 23: 7951–7957. 16258095 10.1200/JCO.2005.01.5479

[7] Giangaspero F Ellison D Eberhart CG Haapasalo H Pietsch T Wiestler OD Pfister S 2016; 198–199.

[8] Goschzik T Zur Mühlen A Kristiansen G Haberler C Stefanits H Friedrich C von Hoff K Rutkowski S Pfister SM Pietsch T Molecular stratification of medulloblastoma: comparison of histological and genetic methods to detect Wnt activated tumours. Neuropathol Appl Neurobiol. 2015; 41: 135–144. 24894640 10.1111/nan.12161

[9] Kaur K Kakkar A Kumar A Purkait S Mallick S Suri V Sharma MC Julka PK Gupta D Suri A Sarkar C Clinicopathological characteristics, molecular subgrouping, and expression of miR-379/miR-656 cluster (C14MC) in adult medulloblastomas. J Neurooncol. epub ahead of print. 2016. 10.1007/s11060-016-2250-6 27576698

[10] Kaur K Kakkar A Kumar A Mallick S Julka PK Gupta D Suri A Suri V Sharma MC Sarkar C Integrating molecular subclassification of medulloblastomas into routine clinical practice: a simplified approach. Brain Pathol. 2015; 26: 334–343. 26222673 10.1111/bpa.12293 PMC8029101

[11] Koch A Hrychyk A Hartmann W Waha A Mikeska T Waha A Schüller U Sörensen N Berthold F Goodyer CG Wiestler OD Birchmeier W Behrens J Pietsch T Mutations of the Wnt antagonist AXIN2 (Conductin) result in TCF-dependent transcription in medulloblastomas. Int J Cancer. 2007; 121: 284–291. 17373666 10.1002/ijc.22675

[12] Koch A Waha A Tonn JC Sörensen N Berthold F Wolter M Reifenberger J Hartmann W Friedl W Reifenberger G Wiestler OD Pietsch T Somatic mutations of WNT/wingless signaling pathway components in primitive neuroectodermal tumors. Int J Cancer. 2001; 93: 445–449. 11433413 10.1002/ijc.1342

[13] Kool M Jones DTW Jäger N Northcott PA Pugh TJ Hovestadt V Piro RM Esparza LA Markant SL Remke M Milde T Bourdeaut F Ryzhova M Sturm D Pfaff E Stark S Hutter S Şeker-Cin H Johann P Bender S Genome sequencing of SHH medulloblastoma predicts genotype-related response to smoothened inhibition. Cancer Cell. 2014; 25: 393–405. 24651015 10.1016/j.ccr.2014.02.004 PMC4493053

[14] Küchler J Hartmann W Waha A Koch A Endl E Wurst P Kindler D Mikeska T Waha A Goodyer CG Büttner R Schilling K Pietsch T p75(NTR) induces apoptosis in medulloblastoma cells. Int J Cancer. 2011; 128: 1804–1812. 20549701 10.1002/ijc.25508

[15] Lamont JM McManamy CS Pearson AD Clifford SC Ellison DW Combined histopathological and molecular cytogenetic stratification of medulloblastoma patients. Clin Cancer Res. 2004; 10: 5482–5493. 15328187 10.1158/1078-0432.CCR-03-0721

[16] Lin CY Erkek S Tong Y Yin L Federation AJ Zapatka M Haldipur P Kawauchi D Risch T Warnatz H-J Worst BC Ju B Orr BA Zeid R Polaski DR Segura-Wang M Waszak SM Jones DTW Kool M Hovestadt V Active medulloblastoma enhancers reveal subgroup-specific cellular origins. Nature. 2016; 530: 57–62. 26814967 10.1038/nature16546 PMC5168934

[17] Louis DNWHO Classification of Tumours of the Central Nervous System Revised 4th Edition. Lyon: International Agency for Research on Cancer. 2016.

[18] McManamy CS Pears J Weston CL Hanzely Z Ironside JW Taylor RE Grundy RG Clifford SC Ellison DW Nodule formation and desmoplasia in medulloblastomas-defining the nodular/desmoplastic variant and its biological behavior. Brain Pathol. 2007; 17: 151–164. 17388946 10.1111/j.1750-3639.2007.00058.x PMC8095556

[19] Northcott PA Korshunov A Witt H Hielscher T Eberhart CG Mack S Bouffet E Clifford SC Hawkins CE French P Rutka JT Pfister S Taylor MD Medulloblastoma comprises four distinct molecular variants. J Clin Oncol. 2011; 29: 1408–1414. 20823417 10.1200/JCO.2009.27.4324 PMC4874239

[20] Pietsch T Ellison DW Haapasalo H Giangaspero F Wiestler OD Eberhart CGDesmoplastic/nodular medulloblastoma. In: Louis DN, Ohgaki H, Wiestler OD, Cavenee WK (eds.) World Health Organization classification of tumours of the central nervous system. Revised 4th edition. IARC. 2016. p.195-197. 2016.

[21] Pietsch T Schmidt R Remke M Korshunov A Hovestadt V Jones DTW Felsberg J Kaulich K Goschzik T Kool M Northcott PA von Hoff K von Bueren AO Friedrich C Mynarek M Skladny H Fleischhack G Taylor MD Cremer F Lichter P Prognostic significance of clinical, histopathological, and molecular characteristics of medulloblastomas in the prospective HIT2000 multicenter clinical trial cohort. Acta Neuropathol. 2014; 128: 137–149. 24791927 10.1007/s00401-014-1276-0 PMC4059991

[22] Remke M Hielscher T Northcott PA Witt H Ryzhova M Wittmann A Benner A von Deimling A Scheurlen W Perry A Croul S Kulozik AE Lichter P Taylor MD Pfister SM Korshunov A Adult medulloblastoma comprises three major molecular variants. J Clin Oncol. 2011; 29: 2717–2723. 21632505 10.1200/JCO.2011.34.9373

[23] Tabori U Baskin B Shago M Alon N Taylor MD Ray PN Bouffet E Malkin D Hawkins C Universal poor survival in children with medulloblastoma harboring somatic TP53 mutations. J Clin Oncol. 2010; 28: 1345–1350. 20142599 10.1200/JCO.2009.23.5952

[24] Taylor MD Northcott PA Korshunov A Remke M Cho Y-J Clifford SC Eberhart CG Parsons DW Rutkowski S Gajjar A Ellison DW Lichter P Gilbertson RJ Pomeroy SL Kool M Pfister SM Molecular subgroups of medulloblastoma: the current consensus. Acta Neuropathol. 2012; 123: 465–472. 22134537 10.1007/s00401-011-0922-z PMC3306779

[25] Zhao F Ohgaki H Xu L Giangaspero F Li C Li P Yang Z Wang B Wang X Wang Z Ai L Zhang J Luo L Liu P Molecular subgroups of adult medulloblastoma: a long-term single-institution study. Neuro-oncol. 2016; 18: 982–990. 27106407 10.1093/neuonc/now050 PMC4896550

[26] Zhukova N Ramaswamy V Remke M Pfaff E Shih DJH Martin DC Castelo-Branco P Baskin B Ray PN Bouffet E von Bueren AO Jones DT Northcott PA Kool M Sturm D Pugh TJ Pomeroy SL Cho YJ Pietsch T Gessi M Subgroup-specific prognostic implications of TP53 mutation in medulloblastoma. J Clin Oncol. 2013; 31: 2927–2935. 23835706 10.1200/JCO.2012.48.5052 PMC4878050

